# A Final Duty on Demobilisation

**Published:** 1919-05-03

**Authors:** 


### THE TEST OF THE RED CROSS.

### A Final Duty on Demobilisation.

In dealing in previous years with the annual report of the Joint Finance Committee of the British Red Cross Society and the Order of St. John of Jerusalem, we have borne in mind the day of reckoning for the management which would arrive with peace, and the need for a policy to direct the expenditure of any surplus that remained in the hands of the Fund. The financial committee adopted a policy known as "the big balance," and the fourth annual report for the year ending on October 18 last brings these questions to the issue. Some light is thrown upon them by the remarks of Sir Robert Hudson on April 1 last, at a special meeting of the Joint War Committee. Last year, he says, the Fund received £20,500 a day and spent £20,000; in other words, 11¾d. were spent out of every shilling received. This, he remarks, can hardly be called hoarding, and its effect was to leave the Fund at the end of the financial year with sufficient in hand to "carry on" for two months. Then came the "Our Day" collection on October 18. It was a piece of good luck that the appeal was made when it was, and that 3¾ millions were collected; for within three weeks the Armistice was declared, and that would probably have defeated any appeal issued simultaneously with it. Even now, he says, the fund is unable to determine what it will be called upon to spend in order to meet its obligations to the sick and wounded; but the report states that "the funds we have in hand will enable us to meet every legitimate demand which we can foresee." So the cessation of appeals on January 1 appears to have been justified.

As to the future, Sir Robert Hudson refuses to consider any claims, until those of the sailors and soldiers—and let us remind him of the staffs which minister to them, from whom equally the money has been subscribed—have been fully met. It is curious that since 1916 the expenditure jumped to 36, and from 36 to 72 successively: these figures represent hundred thousands. The expenditure on stores seems to have taken the lion's share of the disbursements. Since the Armistice, the work of the finance committee has been linked to that of the Demobilisation Board. But of this association the report says little.

We have always tested the virtue of the Fund's management in a humble but direct way. We have asked how it tended members of its own staff, and whether it treated them with the same consideration when in need as that which it expected them to show to their own duties and to the wounded. Therefore the page devoted in the accounts to the Compassionate Fund has assumed an importance for us not to be masked by the more pretentious figures on the pages which catch the eye of the public. It appears from the schedule of this fund that 151 applications for assistance were received in 1918, and that 139 grants were made. More than half the applications came from workers in France and Belgium, but 51 were received from those at home. Motor ambulance men, then orderlies, then V.A.D.s were the groups apparently most in need of assistance. The grants made in 1918 amounted to £2,147, being about £350 more than the total of the grants in 1917. The grants averaged roughly £15 to each applicant, and as the balance of the Compassionate Fund amounts to £21,105, either applications were few, or the grants did not err on the side of generosity. We hope that the existence of the Fund was well known to all the personnel, and that not only was each case generously considered on its merits, but that every person who had a case at all was encouraged to submit it to the Fund. It cannot be too strongly urged that the hospital standard applies equally to the staff as to the patients, and the bare fact that nurses have been admittedly overworked and underpaid shows that this has been forgotten in an indecent haste to reduce expenses. Hospital funds and Red Cross funds, which have a similar origin, exist to be spent rather than saved, and economy means judicious expenditure rather than parsimony.

The multifarious activities of the Fund on every front, in every clime, for every purpose make a brave show, and have been seized on by the lay papers naturally anxious to impress the public. Our aim is rather to apply the hospital standard to the Fund itself, to test its reality at the heart of the whole organisation, and to judge the complete success of the result from that quarter which is least apparent to the public eye. How far the Red Cross could have influenced the salaries, say, of V.A.D.s, to their advantage; how far it bettered the conditions of their work, and saw that this service was recognised generously, it is difficult to say. But it had a great responsibility, a great opportunity, and the reality of its professions must be tested by the degree of the service that it achieved. Now that the demobilisation of Red Cross workers and V.A.D.s has begun apace, sometimes indeed inconsiderately, the Red Cross and especially the Compassionate Fund has still the means somewhat to remedy any past negligence. That which we are concerned to know is the end to which the balance of the Compassionate Fund shall be devoted; for every Red Cross worker and V.A.D. in need should feel that they have a friend in the Compassionate Fund who will see that the transition from war-work to peace work is made easy, and that the Red Cross is as mindful of the loyal service rendered by its workers as it asked them to be of the needs of the sailors and soldiers whom they nursed or cared for.

